# Supplementary material for: Educational Effects of Simulation and Non-Simulation Training in Airway Management according to Levels of the Kirkpatrick Model: A Systematic Review and Network Meta-Analysis
Source: J Clin Med. 2022 Sep 23;11(19):5614. doi: 10.3390/jcm11195614 (PMC9571641; doi:10.3390/jcm11195614)
Supplement: Supplementary file 1 [file jcm-11-05614-s001.zip › jcm-1888044-supplementary.pdf]

Table S1. Characteristics of included studies.

Study	year	N	Type of study	Intervention	Control	Type of non-Sim	Outcome
Abrahamson S	1969	10	RCT	Sim	NI		BpS
Hosking EJ	1998	46	non-RCT	Sim	NI		KnS
Nadel FM	2000	57	non-RCT	Sim	NI		KnS
Naik VN	2001	24	RCT	Sim	non-Sim	Lecture	BpS
Modell JH	2002	40	RCT	Sim	non-Sim	Self-study	KnS
Multak N	2002	56	non-RCT	Sim	non-Sim	Video and discussion	KnS
Morgan PJ	2002	14	RCT	Sim	non-Sim	Video	KnS
Rowe R	2002	20	RCT	Sim	NI		BpS
Noeller TP	2008	38	non-RCT	Sim	NI		KnS
Corbridge SJ	2008	7	non-RCT	Sim	NI		KnS
Gómez LM	2008	29	RCT	Sim	NI		BpS

Wiel E	2009	24	non-RCT	Sim	NI		KnS
Nguyen HB	2009	63	non-RCT	Sim	NI		KnS
Wenk M	2009	33	RCT	Sim	non-Sim	Problem based discussion	KnS
Rubiano AM	2010	37	non-RCT	Sim	NI		KnS
Nishisaki A	2010	78	non-RCT	Sim	NI		BpS
Alyousef	2017	46	non-RCT	Sim	NI		KnS
Han	2018	35	non-RCT	Sim	NI		BpS

N, number of patients; RCT, randomized controlled trial; non-RCT, non-randomized controlled trial; Sim, simulation-based training; non-Sim, non-simulation-based training; I, no educational intervention. In cases where the inclusion studies did not report sufficient data for analysis, the data reported in reference number 13 (which was not included in the inclusion studies) were used in the integrated analysis.
